# Erratum

**DOI:** 10.4269/ajtmh.2011.845err

**Published:** 2011-05-05

In *Am J Trop Med Hyg 83:* 1123–1127 by Hochedez et al, some errors concerning the taxonomic terms appeared:

1. In all the manuscript (including text and tables), the text should read “*Aeromonas* species” instead of “*Aeromonas hydrophila* complex”.
2. In the abstract, the text should read “There have been no reports on *Aeromonas* species in the Caribbean to date.” (*not* “There have been no reports on *Aeromonas hydrophila* complex (*A. hydrophila*, *A. caviae*, *A. veronii*) in the Caribbean to date.”).
3. In the Introduction, text should read “the three species mainly isolated during septicaemia are *A. hydrophila sensu stricto*, *A. veronii* bv. Sobria, and *A. caviae*.” (*not* “the three species mainly isolated during septicaemia belong to the *Aeromonas hydrophila* complex: *A. hydrophila*, *A. veronii*, and *A. caviae*.”)
4. In the Materials and Methods, text should read “Bacteremia caused by *Aeromonas* species were identified by …” (*not* “Bacteremia caused by *A. hydrophila* complex (*A. hydrophila*, *A. veronii*, and *A. caviae*) were identified by ..”)

Additional conventional phenotypic testing would be needed to actually place these clinical isolates into either a species related Complex, e.g. *A. hydrophila* Complex, *A. veronii* Complex, or *A. caviae* Complex or to identify them to the level of an individual species designation. Unfortunately the study was retrospective and most of the *Aeromonas* stains are now unavailable for further identification tests. The authors regret these errors.

